# Grant Peer Review: Improving Inter-Rater Reliability with Training

**DOI:** 10.1371/journal.pone.0130450

**Published:** 2015-06-15

**Authors:** David N. Sattler, Patrick E. McKnight, Linda Naney, Randy Mathis

**Affiliations:** 1 Department of Psychology, Western Washington University, Bellingham, Washington, United States of America; 2 Department of Psychology, George Mason University, Fairfax, Virginia, United States of America; 3 Oak Ridge Associated Universities, Oak Ridge, Tennessee, United States of America; Canadian Agency for Drugs and Technologies in Health, CANADA

## Abstract

This study developed and evaluated a brief training program for grant reviewers that aimed to increase inter-rater reliability, rating scale knowledge, and effort to read the grant review criteria. Enhancing reviewer training may improve the reliability and accuracy of research grant proposal scoring and funding recommendations. Seventy-five Public Health professors from U.S. research universities watched the training video we produced and assigned scores to the National Institutes of Health scoring criteria proposal summary descriptions. For both novice and experienced reviewers, the training video increased scoring accuracy (the percentage of scores that reflect the true rating scale values), inter-rater reliability, and the amount of time reading the review criteria compared to the no video condition. The increase in reliability for experienced reviewers is notable because it is commonly assumed that reviewers—especially those with experience—have good understanding of the grant review rating scale. The findings suggest that both experienced and novice reviewers who had not received the type of training developed in this study may not have appropriate understanding of the definitions and meaning for each value of the rating scale and that experienced reviewers may overestimate their knowledge of the rating scale. The results underscore the benefits of and need for specialized peer reviewer training.

## Introduction

Enhancing reviewer training may improve the reliability and accuracy of research grant proposal scoring [1, 2]. Few studies have examined the efficacy of reviewer training, and “…there is surprisingly little empirically rigorous research” (p. 160) [3] examining peer review [4]. In recent years, some grant funding agencies have required reviewers to watch brief training videos or participate in brief webinar training sessions prior to reviewing proposals. The present authors found no studies assessing the utility of these training programs, examining whether they improve the reliability and consistency of reviewers' scoring of proposals, or examining alternative approaches to increase reliability and scoring accuracy [3, 5].

We designed and evaluated a brief training program that attempts to increase inter-rater reliability and proposal scoring accuracy. Because it is a first step, we focused on specific, fundamental aspects of the grant review process that may influence scoring and that are mutable to some degree: reviewers' knowledge of the review criteria, each value of the rating scale, and the consequences of inaccurate or inappropriate scoring for funding decisions. The training program does not address all potential areas of training or sources of noise that may affect scoring.

Noise can influence the accuracy and reliability of reviewer scoring. Noise involves random error that decreases scoring precision and accuracy. Sources of noise that may influence scoring accuracy and reliability and that may be mutable to some degree during the grant review process include understanding of how to assign scores (e.g., knowledge and application of the grant review criteria and rating scale), familiarity with the grant review process, variability in reviewer knowledge (e.g., match between reviewer's topical knowledge and the proposal content), bias, and extraneous factors [6].

The anchoring bias is a potential source of noise that may occur if reviewers fail to make use of the full scale and instead inappropriately assign scores that are primarily in the upper or lower portion of the scale. This bias can reduce variability and introduce restriction of range problems. The following analogy illustrates one way the anchoring bias may influence a reviewer's selection of rating scale values, and we discuss it in our brief training program and here in some detail. Reviewers who are academics routinely evaluate students' written work and assign grades from A (excellent) to F (fail); they less often use grant review rating scales to evaluate work. There are significant differences between the academic grading model and the scales used to assign scores to grant proposals. In the academic grading model, it is common not to differentiate scores below the 60^th^ percentile because the material receives the same "F" grade (e.g., a paper scored at the 59^th^ percentile receives the same grade as a paper scored at the 10^th^ percentile). In contrast, values and percentages on most grant rating scales do not have equivalent meaning to those of the academic grading model, and they have consequences for the proposal. For example, on the National Institutes of Health (NIH) 9-point grant rating scale (from 1 = exceptional to 9 = poor), a score in the 55% range represents a “good” proposal whereas a score in the 0% to 11% range represents a “poor” proposal. While anchoring bias can occur for several reasons, it can happen if a reviewer inappropriately applies the academic model in selecting a rating scale value. For example, suppose a reviewer believes the proposal deserves a poor score at the 50^th^ percentile based on the academic model (viz., fail), but inappropriately assigns a score close to the mid-way point on the grant rating scale (which would represent a "good" rather than a "poor" proposal). In this case, the score would be inflated and is not accurate; restriction of range would be present. Further, the reviewer might rationalize this value selection by believing that proposals receiving scores in the "good" range would not be funded. This rationalization is similar, to some degree, to the notion that all material evaluated at 59% or below (in the academic grading model) receive a similar outcome. The brief training program in this study instructs reviewers about how potential sources of noise, including the anchoring bias, can influence the scores assigned to proposals as well as the consequences of appropriate and inappropriate scoring.

Since many grant rating scales, including the scale used by NIH, use reverse coding whereby lower numerical values represent more positive ratings, our brief training program also discusses reverse coding and the importance of each value. This information was included to help reduce potential errors associated with scale value selection. Of course, other factors can lead to score inflation and inaccurate score selection. For example, some reviewers may be hesitant to assign an overall negative score due to their unfamiliarity with some portion of proposal content. Other reviewers might want to give the proposal investigators the “benefit of the doubt” that the weaknesses will not compromise the overall goals. Other subjective and motivational factors also may be at work. We did not examine all cognitive biases that may affect the selection of scale values as doing so is beyond the scope of this study.

Given that this is an initial attempt to improve inter-rater reliability, our brief training program focuses on increasing knowledge of the rating scale, knowledge about the consequences of inaccurate scoring, and familiarity with and attention to the grant review criteria. If the program reduces scoring biases, increases the likelihood that raters would correctly select rating scale values, and increases inter-rater reliability, then it would follow that a more systematic evaluation of the effects should continue in this research area. Since experienced reviewers should have a stronger working knowledge and appreciation of the rating scale and grant review criteria, we expected the training program would have greater instructional value for inexperienced rather than experienced reviewers.

## Method

### Participants

The participants were 75 (40 men, 35 women) Public Health professors from research universities across the United States. The average age was 44 years (SD = 10). Most held a Ph.D. (80%), followed by a M.D. (11%), Sc.D. (4%), DPharmacy (2.5%), and both a Ph.D. and M.D. (2.5%). We identified 180 individuals by performing a web-based search for Public Health programs and sent an e-mail soliciting participation. If a response was not received within 1 1/2 weeks, we sent an e-mail or called. They were offered $50 in exchange for their participation.

The project was approved by the ORAU Institutional Review Board and the Western Washington University Human Participants Review Committee. Participants visited a secure website with the study materials that first presented informed consent information. Participants were asked whether they agreed or did not agree with the informed consent information and to click a button indicating their choice. All individuals agreed to participate.

### Design and Procedure

The design was a randomized trial in which participants were randomly assigned to training program condition. Participants in the training program condition visited a secure website that presented informed consent information, introduced the study, presented the training program video, offered participants the option to read the criteria for the funding mechanism, and presented a questionnaire. Participants in the no training program condition also visited a secure website that presented informed consent information, introduced the study, offered the option to read the criteria for the funding mechanism, and presented a questionnaire. Participants did not review research proposals. They merely received criteria consistent with specific rating scale values and were asked to assign ratings that were consistent with those criteria.

Participants also were asked whether they had any extramural grant review experience. Those with no extramural review experience (all Assistant Professors; *N* = 29) were coded as “novices,” and those with experience (Assistant, Associate, and Full Professors; *N* = 46) were coded as “experienced” reviewers. Experienced reviewers had served on an average of 19 extramural review panels (*SD* = 27) whereas novice reviewers had served on no review panels (*M* = 0, *SD* = 0).

Participants completed a questionnaire asking for information about demographics, prior grant review experience, knowledge of the grant review criteria, and understanding of the NIH rating scale. We only used the NIH rating scale because public health professors commonly review proposals using this scale; we could have chosen a rating scale from any of number of federal or private grant funding agencies. We simulated common grant review protocols by asking reviewers to read the grant criteria information; we did not indicate whether we would verify if they had done so.

### Training Video

We produced an 11 minute professional quality training program video that emphasized five fundamental issues: (1) grant agencies depend on reviewers for accurate information; (2) reviewer scores influence funding decisions; (3) the meaning of each value on the NIH 9-point rating scale and the definitions of minor, moderate, and major weakness; (4) how to assign evaluation scores that indicate how well the proposal matches the agency’s criteria; and (5) why it is important to carefully read and understand the agency’s criteria (i.e., how minor differences in scoring can influence funding decisions).

The host stated that the training video was designed to help reviewers evaluate proposals and provide scores that indicate how well the proposals match the agency’s review criteria. To highlight the importance of reviewers’ numerical scores, the host provided an example illustrating how agencies use reviewers’ ratings to reach funding decisions. This example stressed three points: (a) reviewers should carefully read and understand the granting agency’s criteria; (b) reviewers’ evaluation scores should indicate how well the proposal matches the agency’s criteria; and (c) reviewers should understand the rating scale, the meaning of each value of the rating scale, and the definitions of minor, moderate, and major weakness. This example also showed how minor differences in the overall evaluation scores of proposals influence whether or not a proposal scores in the high acceptable range (and hence more likely to be funded). It showed viewers that agencies depend on them for accurate information, that the scores they assign have real consequences, and placed their role as a reviewer in the larger picture of the grant review process.

The host stressed that the rating scale used in the present project may differ from other grant review rating scales as well as rating scales used in other settings and gave an example of those differences. For example, a score in the 50 percent range typically represents a failing score in academia, but on the 9-point scale used in the grant review (viz., NIH rating scale) a value mid-range represents a “good” proposal. The meaning of each scale score (e.g., 1 = a proposal that is exceptionally strong and has essentially no weaknesses; 5 = a good proposal that is strong and has a least one moderate weakness) were discussed in detail. The definitions for degree of weakness as defined by the NIH were discussed (e.g., minor: does not substantially lessen the project’s potential to make a contribution). The video then presented summaries of several proposals with various strengths and weaknesses, asked participants which numerical rating they might assign to each proposal, and presented the correct answer. Finally, the host stressed that reviewers were responsible for carefully reading the grant review criteria and thanked them for participating.

### Questionnaire

The questionnaire asked for the following information.

#### Demographics and Grant Review Experience

Six items asked for demographic information, highest degree earned, and number of extramural and intramural review panels served on. Six items asked about familiarity with state and federal agency grant review criteria and the grant review rating scales used by the NIH and National Science Foundation. Participants used a 5-point scale (1 = not at all to 5 = very much) to indicate their answers. Two questions asked if they read the review criteria presented in this project and how much time they spent doing so.

#### Understanding of Rating Scale

Participants assigned a rating scale score to four proposal summary descriptions that were taken verbatim from the NIH scoring criteria; thus, they serve as a proximal indicator of rating scale knowledge (section in parentheses below are added for the reader’s benefit).

- You believe a proposal is strong and has at least one moderate weakness (*a “Good” proposal that warrants a score of 5*).
- You believe a proposal is strong but has numerous minor weaknesses (*a “Very Good” proposal that warrants a score of 4*).
- You believe a proposal has some strengths but has at least one major weakness (*a “Fair” proposal that warrants a score of 7*)
- You believe a proposal is extremely strong and has negligible weaknesses (*an “Outstanding” proposal that warrants a score of 2*).

Participants could not review the NIH descriptors as they completed the task. We scored each response as correct if it matched the criteria and incorrect if it did not. Accuracy and inter-rater reliability were evaluated separately. Raters were judged to “agree” and were reliable among the group if they used the same rating–regardless of its accuracy. Thus, if all raters indicated a “3” for a single item but the correct response was a “5,” then the raters would agree on an inaccurate response. These responses served as the dependent variables for the inter-rater reliability analyses reported below.

## Results

**Fig 1:**
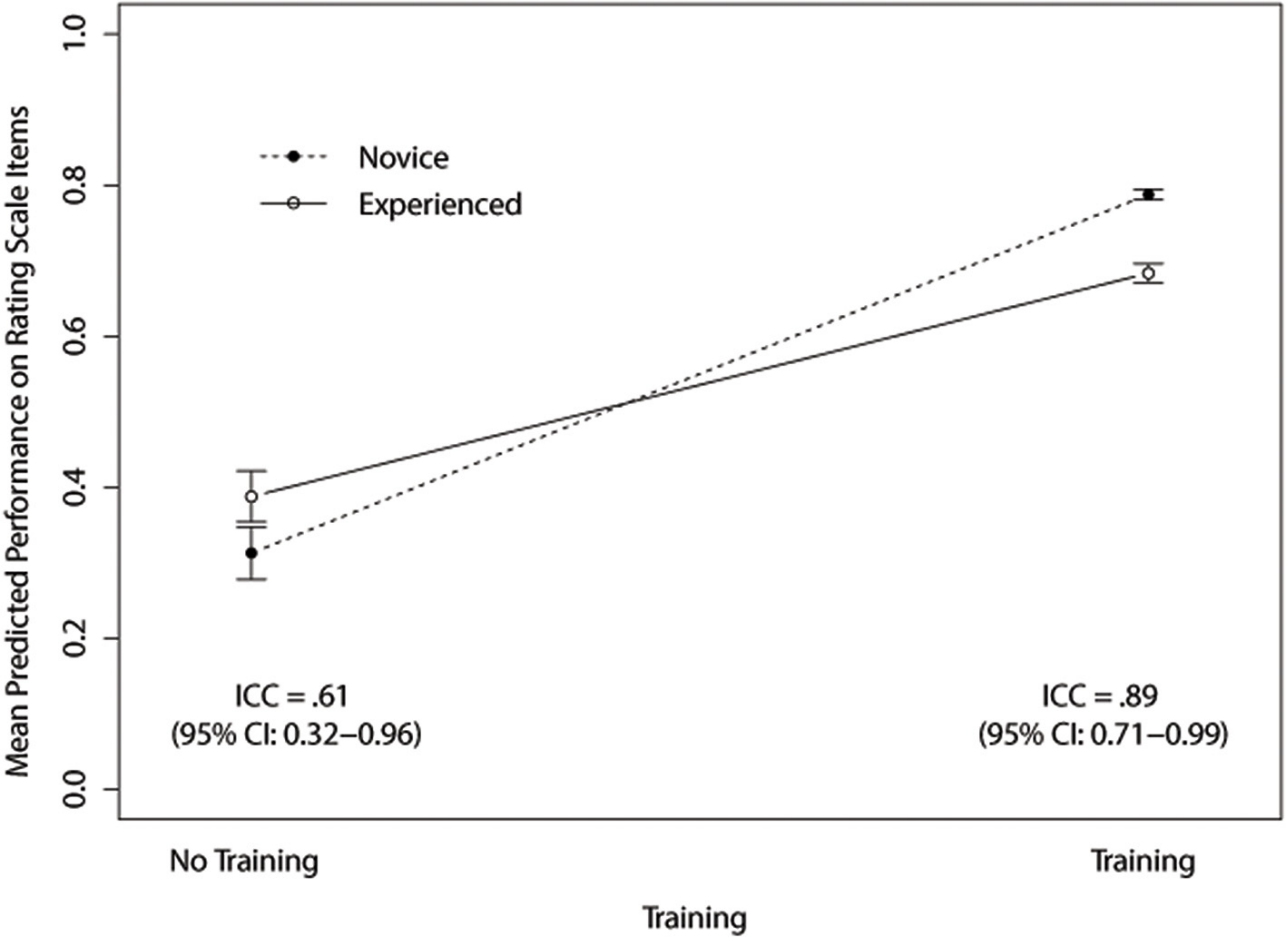
Rating scale knowledge.

The training program significantly improved the correct selection of rating scale values compared to the no training program condition. Participants who received training were more accurate about the rating scale values than those in the no training program condition (74% vs. 35%, respectively, linear regression, *p* < .05; see Fig 1). Reviewer experience had no direct influence and did not interact with the video condition.

To evaluate inter-rater reliability, we chose to compute intra-class correlations (ICC's) instead of alternative measures because they tend to suffer less from high or low agreement and they provide a clearer understanding of the rate of consistency among the raters [7, 8]. The ICC provides an adequate but not essential estimation of inter-rater reliability. Mueller and Buttner provided a good justification and criticism of the ICC with respect to alternative methods [9]. In short, the ICC offers a single metric for inter-rater reliability but the choice of the specific method is essential to getting an adequate estimate that can be interpreted between samples or studies. We chose the ICC with two-way estimation (i.e., items and raters randomly chosen from a bigger pool of persons) for agreement (vs. consistency) and for single values (rather than the average scores across all the items). Shrout and Fleiss described this model as ICC(2,k) or Model B according to Mueller and Buttner [10]. These estimates perform better than the alternatives when more than two raters are used in the study [9].

**Fig 2:**
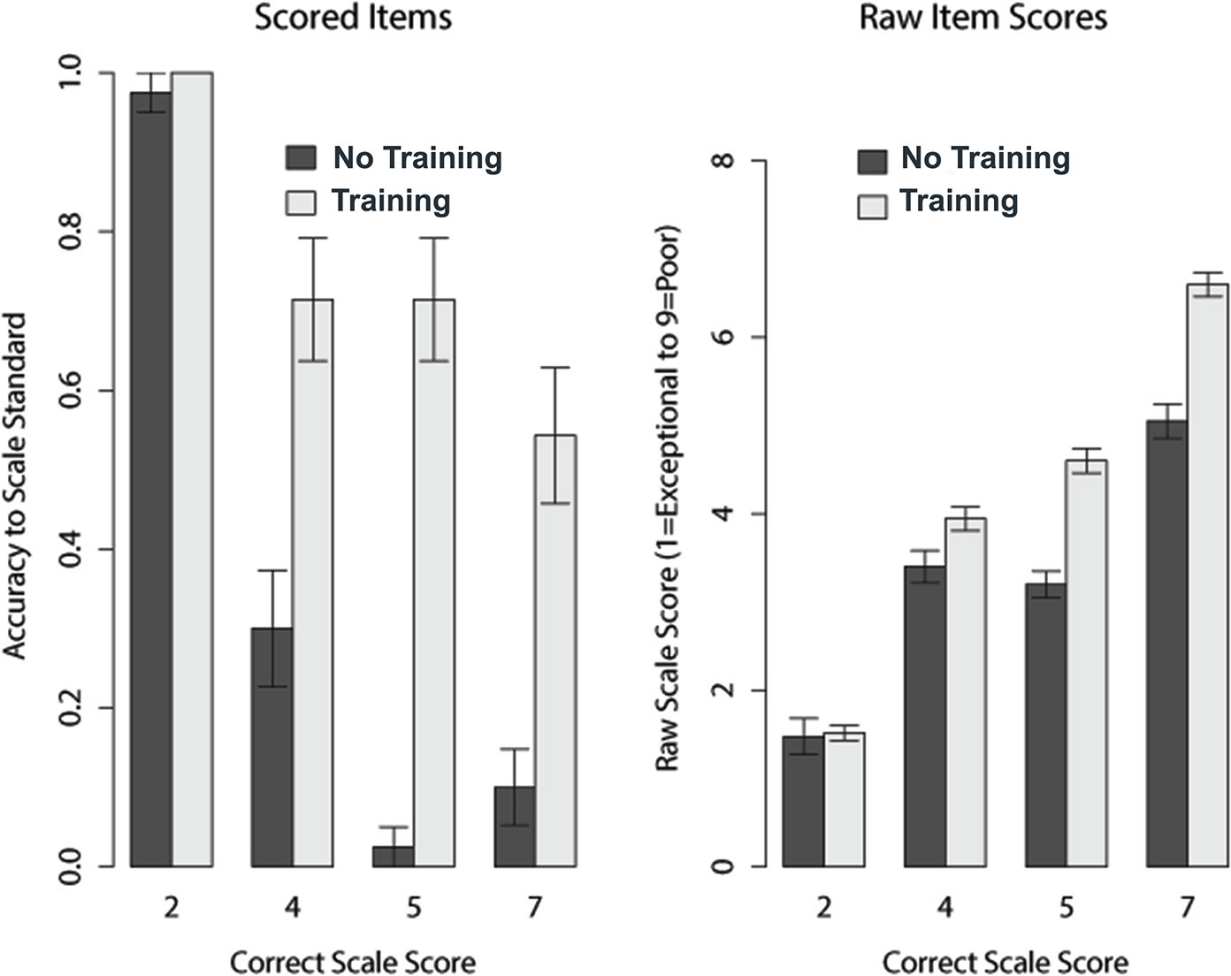
Performance on the four rating scale items (scored and raw items).

Inter-rater reliability was significantly higher in the training program condition (r_icc_ = 0.89; 0.71–0.99 95% CI) than the no training program condition (r_icc_ = 0.61; 0.32–0.96 95% CI; see Fig 1) [10]. Fig 2 shows the effect of training for both the scored rating scale items (i.e., scores based upon the correct response given the rating criteria) and the raw scale scores. Both treatment groups performed well on the scoring task for an “outstanding” proposal (score of 2). However, as the quality of the proposal decreased to very good (score of 4), good (score of 5), and fair (score of 7), the scores of participants in the training program condition were more consistent with the scoring criteria than those in the no training condition. Further, for proposals of lesser quality, both novice and experienced reviewers in the no training condition assigned less accurate and more favorable scores than novice and experienced reviewers in the training program condition.

Participants receiving training spent more time reading the grant review criteria (*M* = 6.1 minutes, *SD* = 4.8) than those in the no training condition (*M* = 4.2 minutes, *SD* = 4.8; Poisson regression, *z* = 2.17, *p* = .03). Additionally, experienced reviewers (M = 6.0 minutes, SD = 5.6) spent more time reading the criteria than the novice reviewers (M = 4.2 minutes, SD = 4.0; Poisson regression, z = 3.22, p = 0.001).

Finally, experienced reviewers reported that they were more familiar with the NIH rating scale (*M* = 4.5, *SD* = .2, where 1 = not at all to 5 = very much) than novice reviewers (*M* = 3.2, *SD* = 1.2), *F*(1,73) = 16.45, *p* = 0001.

There were no age or gender effects in any of the analyses. There were no missing data; participants answered all items.

## Discussion

For both novice and experienced reviewers, our brief training program increased understanding of the rating scale, ability to assign scores that more accurately reflect the rating scale values, and inter-rater reliability. The increase in reliability shown in the training program condition compared to the no training program condition is notable because it is commonly assumed that reviewers—especially those with experience—have good understanding of the meaning of the rating scale. The findings suggest that both experienced and novice reviewers who had not received the type of training developed in this study may not have adequate understanding of the definitions and meaning for each value of NIH rating scales, and that experienced reviewers may overestimate their knowledge of the rating scale.

Both novice and experienced reviewers in the training program condition spent significantly more time reading the review criteria than those in the no training program condition. The training program emphasized that agencies depend on reviewers to indicate how well the proposal matches their criteria and the importance of reading the grant criteria, and asked reviewers to carefully read the grant review criteria. These findings highlight the importance of clearly defining tasks as well as showing their relevance so that reviewers know what is expected of them and why.

There are few studies to which we can compare our results. Our study focused only on rating scale interpretation and application, and any improvements in the use of the rating scale ought to result in improved reliability. Moving beyond the concrete rating scale to the more abstract interpretation of quality and merit would likely decrease inter-rater reliability simply due to differences in reviewer expertise, interest, or preferences. The current participants had limited yet concrete information to evaluate and recall compared to those in the few studies examining inter-rater reliability of full proposals; thus, our results probably reflect a best-case scenario. Despite these differences, our findings are comparable to a degree with these prior studies [11, 12, 5, 4]. Estimates of inter-rater reliability for participants in our no training group were slightly higher (r_icc_ = 0.6) than values reported in these prior studies (.2 < r_icc_ < .4). However, our video trained participants showed higher inter-rater reliability estimates (r_icc_ > .88) than previously reported [13, 14]. The training intervention was designed to be administered just prior to reviewing proposals in order to minimize any recall issues.

Many funding agencies base their decisions, in part, on reviewers' mean proposal ratings. A fundamental assumption underlying these means is that the rating scale represents not just an ordinal rating (i.e., ranking) but also an interval rating (i.e., equal distance between values) where means are informative. Substantial deviations from these interval-level interpretations lead to values that lose their meaning across reviewers and across proposals. Increasing inter-rater reliability and validity of evaluation scores through well designed and empirically tested training programs can promote more accurate scoring and provide agencies with more accurate information to base their funding decisions. When reviewers are not well calibrated [15] with respect to evaluating the relative strengths and weaknesses of proposals, then the reliability and validity of grant reviews may be compromised. Thus, our primary focus in developing the brief training program was the proper use and interpretation of the rating scale.

Several areas of scientific study may pertain to these findings. Educational studies on effective knowledge transfer [16], judgment and decision-making [17], and cognitive neuro-scientifically supported behaviors [18] are relevant to the processes we examined. The fact that we observed significant differences with our brief training intervention ought to open up opportunities to study this process as well as other questions, including the validity of peer review decisions [19] and other factors aside from rating scale usage that can affect inter-rater reliability.

We purposely did not ask reviewers to assess a proposal or judge its merit. This approach allowed us to focus on a narrow aspect of the review process to examine whether training can influence the accuracy of score selection and improve inter-rater reliability apart from proposal content. As such, to some degree this study assessed exposure to and memory of the criteria; both are vital aspects of the process we examined. We did not address other aspects of the review process, such as motivation to encode the information or attend to subtle differences not emphasized in the training, or the desire to adhere to the criteria.

Many grant agencies require reviewers to rate proposals using various criteria, such as significance, investigator qualifications, innovation, approach, and environment [20]. Addressing each one of these criteria in addition to factors affecting reviewer reliability and validity would require a huge research program rather than a single study. Instead, we focused on a single part that represents a starting point–the rating scale usage. Future research may build upon this study by conducting small, targeted interventions such as this brief training program to determine their effectiveness in improving reliability and validity. Research also may examine how reviewer values or preferences may influence decisions, as well as how discussion among reviewers at panel meetings influence proposal ratings.
